# Protective Effects of a Propolis–*Petasites japonicus* Mixture on Scopolamine-Induced Memory Impairment in Mice

**DOI:** 10.4014/jmb.2602.02019

**Published:** 2026-04-27

**Authors:** Mi Yeung Kim, Jae Young Shin, Jun Hyeong Lim, Ji Hyeon Park, Yong Kap Hur, Soon-Il Yun, Byoung Ok Cho, Seon Il Jang

**Affiliations:** 1 Research Institute, Unique Biotech Co., Ltd, Jeonbuk-do 54576, Republic of Korea; 2 Department of Food Science and Technology, Jeonbuk National University, Jeonbuk-do 54896, Republic of Korea; 3 Institute of Health Science, Jeonju University, Jeonbuk-do 55069, Republic of Korea; 4 Department of Health Management, Jeonju University, Jeonbuk-do 55069, Republic of Korea

**Keywords:** Memory impairment, Cholinergic dysfunction, Neuroinflammation, BDNF–TrkB signaling, Propolis–*Petasites japonicus* mixture

## Abstract

Memory impairment associated with cholinergic dysfunction is a key feature of cognitive decline. Natural products with neuroprotective properties have attracted increasing interest as potential interventions for memory dysfunction. This study investigated the protective effects of a propolis–*Petasites japonicus* mixture (PPJM) against scopolamine-induced memory impairment in mice and explored its underlying mechanisms. Male C57BL/6 mice were pretreated with PPJM prior to scopolamine administration. Spatial learning and memory were evaluated using the Morris water maze. Hippocampal cholinergic function was assessed by measuring acetylcholinesterase (AChE) and choline acetyltransferase (ChAT) activities. Molecular mechanisms were examined by Western blot analysis of neuroplasticity-related signaling (BDNF–TrkB–AKT–CREB), phosphorylated Tau (p-Tau), MAPK activation, and neuroinflammatory markers. Immunohistochemical staining for TrkB and p-Tau, along with histological analysis, was performed to assess hippocampal alterations. PPJM pretreatment significantly ameliorated scopolamine-induced deficits in spatial learning and memory, as indicated by reduced escape latency and increased time spent in the target quadrant. PPJM partially restored cholinergic function by reducing AChE activity and increasing ChAT activity in the hippocampus. In addition, PPJM was associated with increased BDNF–TrkB–AKT–CREB signaling, along with reduced p-Tau and attenuation of JNK/p38 MAPK activation. Neuroinflammatory responses, including NF-κB activation and the expression of COX-2, TNF-α, and IL-6, were also reduced following PPJM pretreatment. Histological analyses further indicated preservation of neuronal architecture and modulation of TrkB and p-Tau expression in the hippocampus. These findings suggest that PPJM exerts protective effects against scopolamine-induced memory impairment, accompanied by modulation of cholinergic function, neuroplasticity-related signaling, and neuroinflammatory responses. However, further studies are required to validate these effects in chronic and translational models.

## Introduction

Cognitive impairment is a core symptom commonly observed during aging and in various neurodegenerative disorders, with dysfunction of the cholinergic neurotransmission system recognized as a major pathophysiological contributor to learning and memory deficits [1, 2]. Scopolamine, a muscarinic acetylcholine receptor antagonist, disrupts hippocampus-dependent memory formation and is therefore widely used as a representative experimental model of cognitive impairment [3]. In addition to inducing cholinergic dysfunction, scopolamine administration has been reported to be accompanied by multiple molecular alterations, including reduced neuroplasticity, increased oxidative stress, activation of inflammatory responses, and tau protein phosphorylation [4, 5].

In recent years, increasing attention has been directed toward natural product–derived materials as therapeutic strategies for improving cognitive function [6]. Natural products contain diverse bioactive compounds and possess the potential to modulate multiple pathological pathways simultaneously, rather than targeting a single molecular mechanism [7]. In particular, oxidative stress and neuroinflammation are closely associated with cognitive decline, and natural products with antioxidant and anti-inflammatory properties have attracted considerable interest in preclinical studies due to their potential neuroprotective and cognition-enhancing effects [8]. For example, polyphenols and flavonoid compounds have been reported to contribute to the suppression of neuronal cell death, regulation of synaptic plasticity, and recovery of memory function through their antioxidant and anti-inflammatory activities [9].

Propolis is a resinous substance collected by honeybees from plant exudates and is a complex mixture rich in flavonoids, phenolic compounds, and other bioactive constituents. Numerous studies have demonstrated its potent antioxidant and anti-inflammatory properties [10]. Beyond its well-documented cardioprotective, anticancer, and immunomodulatory effects, the biological activities of propolis have increasingly been extended to neuroprotection, leading to active investigations of its efficacy in experimental models of cognitive impairment [11]. Meanwhile, *Petasites japonicus* has traditionally been used as a medicinal plant for the treatment of inflammatory conditions, and accumulating evidence indicates that extracts derived from its aerial parts and roots exhibit anti-inflammatory, antioxidant, and cytoprotective activities [12]. Notably, *P. japonicus* extracts have been shown to reduce tissue damage by suppressing pro-inflammatory cytokine production and alleviating oxidative stress, suggesting their potential influence on stress-responsive and inflammatory signaling pathways in the nervous system [13].

In our previous study, we optimized the mixing ratio of a propolis and *P. japonicus* combination, referred to as the propolis–*P. japonicus* mixture (PPJM), and verified its pronounced antioxidant and anti-inflammatory activities [14, 15]. These findings suggest that PPJM may exert protective effects against cognitive impairment associated with neuroinflammation and oxidative stress.

However, the protective effects of PPJM on cognitive dysfunction and the underlying molecular mechanisms have not yet been fully elucidated. Therefore, the present study aimed to evaluate the effects of PPJM pretreatment on learning and memory performance in a scopolamine-induced cognitive impairment mouse model and to investigate its protective mechanisms, focusing on cholinergic neurotransmission, neuroplasticity-related signaling pathways, and inflammation- and stress-associated molecular responses.

## Materials and Methods

### Reagents

5,5'-Dithiobis(2-nitrobenzoic acid) (DTNB; D218200) and acetylcholine iodide (A7000) were purchased from Sigma-Aldrich (USA). RIPA buffer (#89901), Halt™ Protease and Phosphatase Inhibitor Cocktail (#78440), goat anti-rabbit IgG secondary antibody (#31460), goat anti-mouse IgG secondary antibody (#31430), and p-TrkB antibody (PA5-36695) were obtained from Thermo Fisher Scientific (USA). Antibodies against IL-6 (#12912), BDNF (#60071), p-Akt (#4060), Akt (#9272), p-CREB (#9198), CREB (#9197), p-Tau (#12885), and β-actin (#4970) were purchased from Cell Signaling Technology (USA). Antibodies against TrkB (sc-377218), p-NF-κB (sc-8008), NF-κB (sc-166748), p-JNK (sc-6254), JNK (sc-7345), p-p38 (sc-7973), p38 (sc-81621), Tau (sc-32274), and TNF-α (sc-52746) were obtained from Santa Cruz Biotechnology (USA). The COX-2 antibody (160126) was purchased from Cayman Chemical (USA). An H&E staining kit was purchased from Abcam (UK). WestGlow™ FEMTO was purchased from Biomax (Republic of Korea). The ImmPRESS^®^ HRP Universal IHC Kit was obtained from Vector Laboratories (USA). A Choline Acetyltransferase Assay Kit (MBS2548424) was purchased from MyBioSource, Inc. (USA).

### Preparation of *P. japonicus* Leaf Extract and Propolis Extract

Leaves of *P. japonicus* were collected in August 2024 from Cheonjam Mountain, Jeonju, Jeollabuk-do, Republic of Korea. The plant material was taxonomically identified by a specialist, and a voucher specimen (No. 2024-08-01) was deposited at the laboratory of the Department of Healthcare & Science, Jeonju University. The collected leaves were washed approximately five times with distilled water and air-dried in a well-ventilated shaded area. The dried leaves (100 g) were extracted with 70% (v/v) ethanol (2 L) at room temperature for 48 h. The extract was filtered through a 0.5 μm membrane filter (ADVANTEC), and the filtrate was concentrated under reduced pressure at 45°C using a rotary evaporator (EYELA N-1100). The concentrate was subsequently dried to obtain a powdered extract. Propolis extract was provided by Unique Biotech Co., Ltd. (Republic of Korea). Briefly, L-arginine and distilled water were mixed at a ratio of 1:2.5 (w/v) and stirred at room temperature for 1 h. Ground propolis was added to the mixture and extracted at room temperature for 4 h. After standing for 16 h, the extract was filtered using a filtration system and dried to obtain a powdered extract. The propolis extract and *P. japonicus* leaf extract were mixed at a ratio of 1:1 (w/w) to prepare the PPJM.

### Animals and Experimental Design

Eight-week-old male C57BL/6J mice were obtained and housed under standard laboratory conditions (12 h light/dark cycle, 24 ± 1°C, relative humidity 55 ± 10%) with free access to food and water. The doses of PPJM (50 and 200 mg/kg) were selected based on previously reported pharmacological studies of propolis and *Petasites japonicus* extracts in rodent models, in which doses ranging from approximately 50 to 300 mg/kg have been widely used to evaluate biological activity without apparent toxicity. Accordingly, 50 mg/kg and 200 mg/kg were chosen to represent low and high pharmacologically relevant doses in the present study. Mice were randomly assigned to five groups: normal control, scopolamine-treated control, PPJM low-dose (50 mg/kg), PPJM high-dose (200 mg/kg), and donepezil-treated (3 mg/kg) groups (n = 6 per group, total n = 30). Each “n” represents an independent biological replicate (individual animal). After one week of acclimatization, oral administration was initiated once daily. PPJM or donepezil was administered throughout the experimental period. Scopolamine (2 mg/kg, *i.p.*) was administered to all groups except the normal control group 30 min prior to each Morris water maze test session. All animal experimental procedures were approved by the Institutional Animal Care and Use Committee (IACUC) of Jeonju University (Approval No. jjIACUC-20250904-2025-0904-A1) and were conducted in accordance with the National Institutes of Health guidelines for the care and use of laboratory animals.

### Morris Water Maze Test

Spatial learning and memory were assessed using the Morris water maze test. The apparatus consisted of a circular pool (100 cm in diameter, 50 cm in height) filled with water (22 ± 1°C) rendered opaque with white food coloring and divided into four equal quadrants. A circular acrylic platform (6 cm in diameter, 29 cm in height) was submerged 1 cm below the water surface and placed at the center of the northwest quadrant. Animal movements were recorded and analyzed using a video tracking system (Smart Junior, Panlab SL, Spain). Behavioral testing and data analyses were performed by investigators blinded to the treatment groups. Prior to training, mice were placed on the platform for 10 s to familiarize them with its location. Acquisition trials were conducted for four consecutive days, during which escape latency and cumulative path length were recorded. On day 5, a probe trial was performed for 120 s with the platform removed, and the time spent in the target quadrant was measured to evaluate spatial memory retention. After each trial, mice were dried under an infrared lamp before being returned to their home cages. At the end of the behavioral tests, mice were anesthetized with isoflurane and sacrificed by cervical dislocation, and hippocampal tissues were rapidly collected.

### Measurement of Acetylcholinesterase and Choline Acetyltransferase Activities

Bilateral hippocampi were rapidly dissected and homogenized in 0.1 M phosphate buffer (pH 8.0) at a ratio of 1:10 (w/v). The homogenates were centrifuged at 1,000 rpm for 10 min at 4°C, and the supernatants were collected. For acetylcholinesterase (AChE) activity, the reaction mixture consisted of 33 μL of supernatant, 470 μL of 0.1 M phosphate buffer (pH 8.0), 167 μL of DTNB (3 mM), and 280 μL of acetylcholine iodide (1 mM). Absorbance was measured at 412 nm, and AChE activity was calculated using the Beer–Lambert law with an extinction coefficient of 13.6 mM^−1^·cm^-1^ and expressed as units per milligram of protein. Protein concentrations were determined using the Bradford method. Choline acetyltransferase (ChAT) activity was measured according to the manufacturer’s instructions.

### Western Blot Analysis

Following the Morris water maze test, hippocampal tissues (20–40 mg) were homogenized in RIPA buffer containing a protease and phosphatase inhibitor cocktail. After incubation at 4°C for 30 min, homogenates were centrifuged at 12,000 rpm for 15 min, and the supernatants were collected. Protein concentrations were quantified using the Bradford assay and normalized with distilled water. Equal amounts of protein (50 μg) were mixed with 5× SDS loading buffer, separated by 10% SDS–PAGE, and transferred onto PVDF membranes. Membranes were blocked with 5% skim milk in TBST for 1 h at room temperature and incubated overnight at 4°C with primary antibodies. After washing, membranes were incubated with HRP-conjugated secondary antibodies for 2 h at room temperature. Protein bands were visualized using WestGlow™ FEMTO chemiluminescent substrate and imaged using a chemiluminescence detection system. Densitometric analysis was performed using ImageJ software (NIH, USA). Band intensities were quantified from the original images by measuring the integrated density of each band within a defined region of interest. The values were normalized to the corresponding loading control (β-actin) or to the respective total protein for phosphorylated targets. All analyses were performed using independent biological samples from individual animals (n = 6 per group).

### Hematoxylin and Eosin Staining

Paraffin-embedded brain sections were deparaffinized in xylene and rehydrated through graded ethanol solutions (100%, 95%, 80%, and 70%) to distilled water. Sections were stained with Mayer’s hematoxylin for 2 min, washed with distilled water, and counterstained with eosin Y for 2 min. After dehydration and clearing through graded ethanol and xylene, sections were mounted with Canada balsam and examined under a light microscope.

### Immunohistochemical Analysis

Paraffin-embedded sections were deparaffinized and rehydrated as described above. Antigen retrieval was performed by heating sections in citrate buffer (pH 6.0) for 30 min. Endogenous peroxidase activity was quenched with 1% hydrogen peroxide for 10 min. After washing, sections were blocked with 2.5% normal horse serum for 1 hours at room temperature and incubated overnight at 4°C with primary antibodies against p-TrkB and p-Tau. Immunoreactivity was detected using the ImmPRESS HRP Universal PLUS Polymer Kit according to the manufacturer’s instructions, followed by visualization with NovaRED and DAB substrates. Sections were counterstained with Mayer’s hematoxylin, dehydrated, cleared, mounted, and analyzed under a microscope. Quantitative analysis of immunoreactivity was performed using ImageJ software (NIH). DAB signals were isolated by color deconvolution, and integrated density values were measured after background subtraction. Relative staining intensity was calculated by normalizing the corrected values to the normal control group (set to 100%). For quantitative analysis, three hippocampal sections per animal were analyzed. Regions of interest (ROI) corresponding to the CA1 area were defined according to anatomical landmarks of the mouse hippocampus. All image analyses were performed using ImageJ software by an investigator blinded to the experimental groups.

### Chemical characterization of PPJM

To ensure the identity and reproducibility of the test material, PPJM was characterized by HPLC analysis. Representative marker compounds derived from the raw materials were selected for quantification, including pinocembrin and chrysin from propolis and bakkenolide B from *Petasites japonicus*. Quantitative analysis was performed using external standard calibration curves. Detailed chromatographic profiles and batch-to-batch comparison data are provided in the Supplementary Materials.

### Statistical Analysis

All statistical analyses were performed using SPSS version 30.0 (IBM, USA). Data are presented as the mean ± standard deviation (SD). Escape latency during the acquisition phase of the Morris water maze test (days 1–4) was analyzed using two-way repeated-measures ANOVA with training day as the within-subject factor and treatment group as the between-subject factor. Mauchly's test of sphericity indicated a violation of the sphericity assumption (W = .436, *p* = .001); therefore, degrees of freedom were corrected using the Greenhouse–Geisser estimate (ε = .665). When significant interactions were detected, post hoc comparisons were performed using TBonferroni’s post hoc test. Path length on the fourth training day, probe trial parameters, and biochemical analyses were analyzed using one-way ANOVA followed by Tukey’s post hoc test. A *p*-value < 0.05 was considered statistically significant.

## Results

### Chemical Characterization of PPJM

HPLC analysis confirmed the presence of the characteristic marker compounds pinocembrin, chrysin, and bakkenolide B in PPJM. Quantitative analysis showed comparable levels of these compounds across three independent production batches, indicating good batch-to-batch consistency of the mixture. Representative chromatograms and quantitative data are presented in the Supplementary Materials (Fig. S1 and Table S1).

### PPJM Pretreatment Ameliorates Scopolamine-Induced Spatial Learning and Memory Impairment

**Fig. 1:**
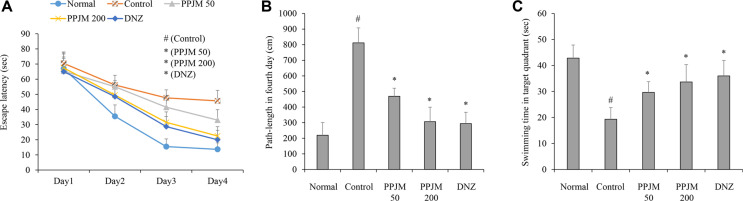
Effects of PPJM pretreatment on scopolamine-induced spatial learning and memory impairment in mice. Spatial learning and memory were evaluated using the Morris water maze test. (**A**) Escape latency during the acquisition trials (days 1–4). (**B**) Path length on training day 4. (**C**) Time spent in the target quadrant during the probe trial. Data are presented as mean ± SD (n = 6 per group). Escape latency (**A**) was analyzed using two-way repeated-measures ANOVA (with Greenhouse–Geisser correction) followed by Bonferroni’s post hoc test for between-group comparisons at each training day. Path length (**B**) and probe trial data (**C**) were analyzed using one-way ANOVA followed by Tukey’s post hoc test. #*p* < 0.05 vs. Normal group; * *p* < 0.05 vs. Scopolamine-treated control group.

Scopolamine administration significantly increased escape latency during the acquisition phase compared with the normal control group. Two-way repeated-measures ANOVA revealed significant main effects of Day (F(2.00, 49.91) = 182.34, *p* < .001), Group (F(4, 25) = 21.05, *p* < .001), and a Day × Group interaction (F(7.99, 49.91) = 3.93, *p* = .001). Due to the violation of sphericity, the Greenhouse–Geisser correction (epsilon = 0.665) was applied to the degrees of freedom. These results indicate significant differences in learning performance among groups over time. PPJM pretreatment significantly attenuated scopolamine-induced impairments in spatial learning (Fig. 1A). Subsequent Bonferroni’s post hoc tests confirmed that PPJM significantly reduced escape latency compared with the scopolamine-treated group at specific training days (*e.g.*, Days 3 and 4). Consistent with these findings, scopolamine significantly increased the path length on the fourth training day, whereas PPJM pretreatment significantly reduced it (*p* < .001; Fig. 1B). In the probe trial, PPJM pretreatment significantly increased the time spent in the target quadrant relative to the scopolamine-treated control group (*p* = .002; Fig. 1C). No significant differences in swim speed were observed (Fig. S2), suggesting that the improvements were not due to locomotor changes.

### Restoration of Hippocampal Cholinergic Function by PPJM in Scopolamine-Induced Memory Impairment

**Fig. 2:**
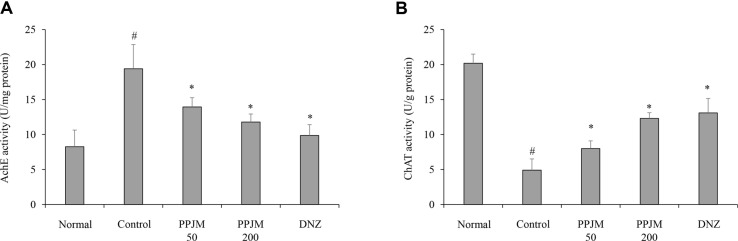
Effects of PPJM pretreatment on hippocampal cholinergic dysfunction induced by scopolamine. Cholinergic function was assessed by measuring (**A**) acetylcholinesterase (AChE) activity and (**B**) choline acetyltransferase (ChAT) activity in hippocampal tissue. Each bar represents the mean ± SD (n = 6). ^#^*p* < 0.05 vs. Normal group. *p < 0.05 vs. Control group.

Scopolamine administration resulted in a significant increase in acetylcholinesterase (AChE) activity and a concomitant decrease in choline acetyltransferase (ChAT) activity in the hippocampus compared with the normal control group. In contrast, PPJM pretreatment significantly and dose-dependently reduced AChE activity compared with the scopolamine-treated control group, with a maximal reduction of approximately 49% observed at 200 mg/kg (*p* = 0.035; Fig. 2A). Similarly, ChAT activity was significantly restored by PPJM pretreatment in a dose-dependent manner, showing an increase of up to approximately 65% at the highest dose compared with the scopolamine-treated control group (*p* = 0.021; Fig. 2B).

### PPJM Activates the Hippocampal BDNF–TrkB–AKT–CREB Signaling Pathway

**Fig. 3:**
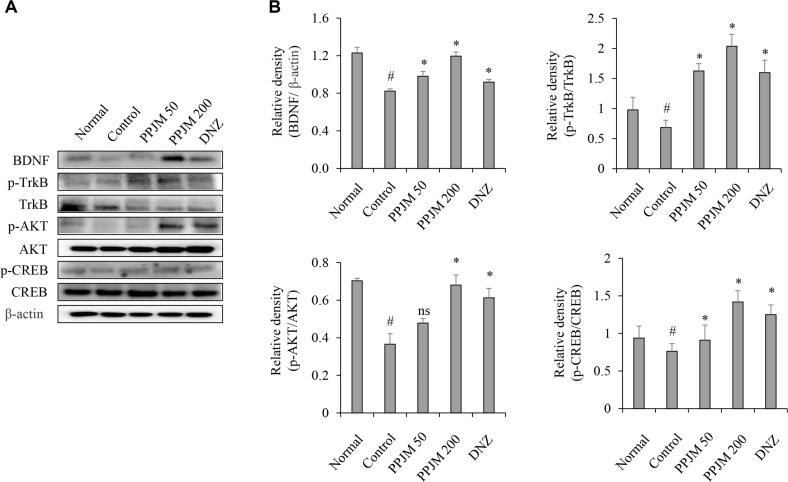
PPJM pretreatment activates the hippocampal BDNF–TrkB–AKT–CREB signaling pathway in scopolamine-treated mice. (**A**) Representative western blot images of BDNF, p-TrkB, TrkB, p-AKT, AKT, p-CREB, and CREB in hippocampal tissue. (**B**) Quantitative analysis of relative band intensity of BDNF (normalized to β-actin), p-TrkB, p-AKT, and p-CREB (each normalized to their respective total protein). Each bar represents the mean ± SD (n = 6). ^#^*p* < 0.05 vs. Normal group. *p < 0.05 vs. Control group. ns, no significant difference compared to Control group.

To investigate the effects of PPJM on neuroplasticity-related signaling, the BDNF–TrkB–AKT–CREB pathway in hippocampal tissue was analyzed by Western blotting. Scopolamine administration significantly reduced BDNF expression and TrkB phosphorylation levels compared with the normal control group (*p* < 0.05). PPJM pretreatment markedly counteracted these effects, significantly increasing BDNF expression and TrkB phosphorylation, with increases of approximately 50% and 194%, respectively, at 200 mg/kg compared with the scopolamine-treated control group (*p* = 0.002 and *p* < 0.001, respectively; Fig. 3). Consistent with TrkB activation, the phosphorylation levels of downstream signaling molecules AKT and CREB, which were suppressed by scopolamine administration, were significantly and dose-dependently restored by PPJM pretreatment (*p* < 0.05; Fig. 3).

### PPJM Suppresses Tau Hyperphosphorylation and MAPK Signaling Activation

**Fig. 4:**
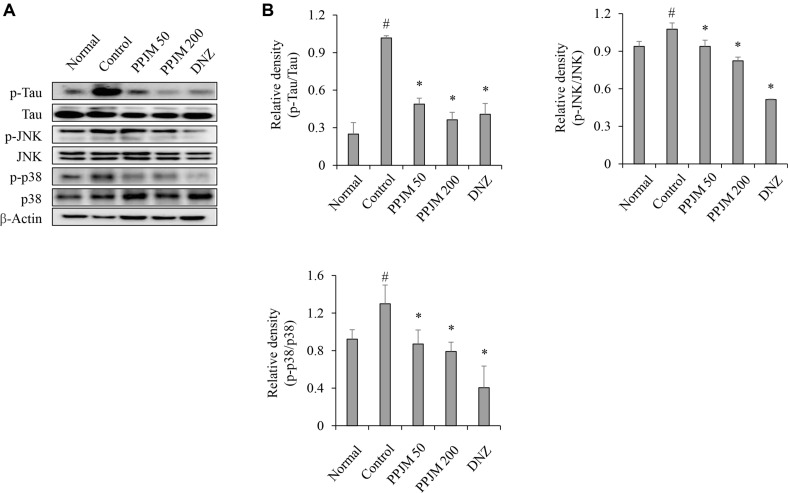
Effects of PPJM pretreatment on p-Tau and MAPK signaling in the hippocampus of scopolamine-treated mice. (**A**) Representative western blot images of p-Tau, tau, p-JNK, JNK, p-p38, and p38. (**B**) Quantitative analysis of relative band intensity of p-Tau, p-JNK, and p-p38. Each bar represents the mean ± SD (n = 6). ^#^*p* < 0.05 vs. Normal group. *p < 0.05 vs. Control group.

To further characterize the molecular pathological changes associated with scopolamine-induced cognitive impairment, p-Tau and MAPK signaling pathways were examined in the hippocampus. Scopolamine administration significantly increased p-Tau levels compared with the normal control group. In addition, phosphorylation of stress-activated MAPKs, including JNK and p38, was markedly enhanced following scopolamine treatment. PPJM pretreatment significantly and dose-dependently reduced p-Tau levels, resulting in a reduction of approximately 64% at 200 mg/kg compared with the scopolamine-treated control group. Furthermore, PPJM pretreatment significantly attenuated the scopolamine-induced activation of p-JNK and p-p38 (*p* = 0.003; Fig. 4).

### PPJM Attenuates Scopolamine-Induced Neuroinflammatory Mediators and NF-κB Signaling

**Fig. 5:**
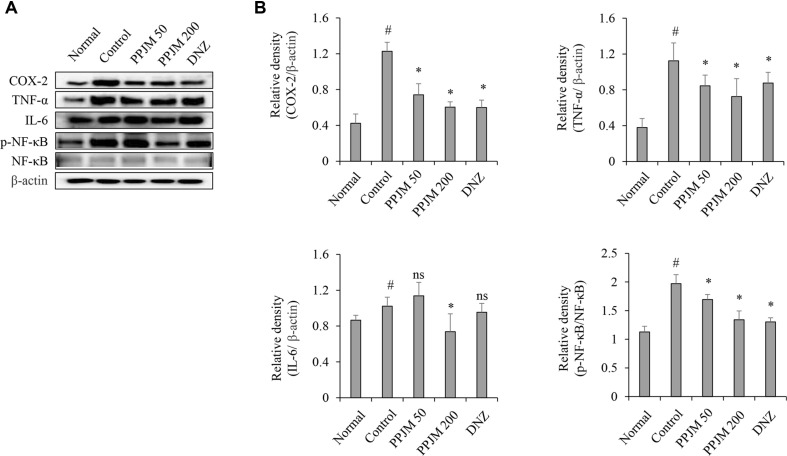
PPJM pretreatment attenuates scopolamine-induced neuroinflammatory signaling in the hippocampus. (**A**) Representative western blot images of p-NF-κB, NF-κB, COX-2, TNF-α, and IL-6. (**B**) Quantitative analysis of relative band intensity of phosphorylated NF-κB (normalized to total NF-κB) and COX-2, TNF-α, and IL-6 (normalized to β-actin). Each bar represents the mean ± SD (n = 6). ^#^*p* < 0.05 vs. Normal group. *p < 0.05 vs. Control group. ns, no significant difference compared to Control group.

To assess the effects of PPJM on neuroinflammatory responses, the expression levels of COX-2, TNF-α, and IL-6, as well as the activation of NF-κB, were evaluated in hippocampal tissue. As shown in Fig. 5, scopolamine administration significantly increased the protein expression of COX-2 and TNF-α compared with the normal control group, while IL-6 levels showed an increasing trend. PPJM pretreatment significantly reduced the elevated expression of COX-2 and TNF-α (*p* < 0.05) induced by scopolamine administration. In contrast, a statistically significant reduction in IL-6 expression was observed only in the PPJM 200 mg/kg group compared with the scopolamine-treated control group (*p* < 0.001). In parallel, scopolamine administration significantly enhanced NF-κB phosphorylation, whereas PPJM pretreatment dose-dependently suppressed NF-κB activation (*p* = 0.04).

### PPJM Preserves Hippocampal Neuronal Integrity and Modulates TrkB and p-Tau Expression

**Fig. 6:**
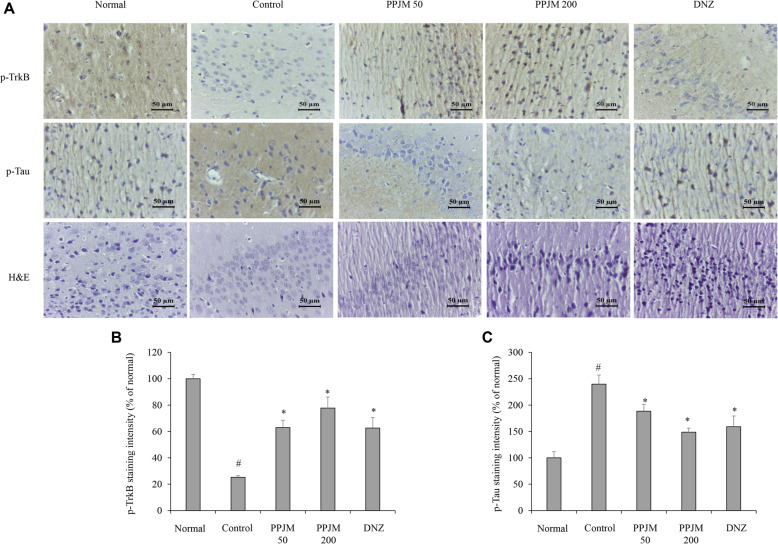
Immunohistochemical and histological analysis of hippocampal tissue following PPJM pretreatment in scopolamine-treated mice. (**A**) Representative immunohistochemical images of p-TrkB and p-Tau, and H&E-stained hippocampal sections. (**B**) Quantification of p-TrkB staining intensity. (**C**) Quantification of p-Tau staining intensity. Scale bar, 200 μm. Each bar represents the mean ± SD (n = 6). ^#^*p* < 0.05 vs. Normal group. *p < 0.05 vs. Control group.

Histopathological alterations and protein expression patterns in the hippocampal CA1 region were examined using immunohistochemical staining for TrkB and p-Tau, as well as H&E staining (Fig. 6). In the scopolamine-treated control group, TrkB immunoreactivity was markedly reduced compared with the normal control group. PPJM pretreatment significantly restored TrkB-positive staining intensity, with more pronounced effects observed at 200 mg/kg (*p* < 0.001). Conversely, scopolamine administration resulted in a substantial increase in p-Tau immunoreactivity in the hippocampus, which was noticeably attenuated by PPJM pretreatment (*p* = 0.002). H&E staining revealed irregular neuronal arrangement and cellular shrinkage in the CA1 region following scopolamine administration, whereas PPJM pretreatment alleviated these histological abnormalities and preserved neuronal morphology comparable to that of the normal control group.

## Discussion

In the present study, we demonstrated that pretreatment with a PPJM effectively attenuates scopolamine-induced cognitive impairment through the coordinated modulation of cholinergic neurotransmission, neuroplasticity-related signaling, tau pathology, stress-activated MAPK pathways, and neuroinflammatory responses in the hippocampus. These findings provide convergent behavioral, biochemical, molecular, and histological evidence supporting the neuroprotective effects of PPJM in a pharmacologically induced memory impairment model.

The chemical composition of PPJM was analyzed using HPLC–DAD, and three representative marker compounds were identified: pinocembrin, chrysin, and bakkenolide B (Fig. S1 and Table S1). These compounds are known to possess neuroprotective and anti-inflammatory activities. Flavonoids derived from propolis, such as pinocembrin and chrysin, have been reported to exert antioxidant and neuroprotective effects in experimental models of neurodegenerative diseases [16, 17], whereas bakkenolide B derived from *Petasites japonicus* has been associated with anti-inflammatory and neuroprotective properties [13]. Although the present study did not determine the individual contribution of each compound, these bioactive constituents may collectively contribute to the neuroprotective effects of PPJM.

Behavioral assessments using the Morris water maze revealed that scopolamine administration markedly impaired spatial learning and memory, as evidenced by prolonged escape latency and increased path length during acquisition trials, as well as reduced time spent in the target quadrant during the probe trial. PPJM pretreatment significantly ameliorated these deficits, indicating a significant protective effect against scopolamine-induced disruption of hippocampus-dependent spatial cognition. Importantly, the improvement observed during the probe trial suggests enhanced spatial memory retention. Consistent with this interpretation, no significant differences in swim speed were observed among the experimental groups, indicating that the behavioral improvements were unlikely to be attributable to alterations in locomotor performance.

Cholinergic dysfunction is a well-established contributor to cognitive impairment in both Alzheimer’s disease and scopolamine-induced amnesia models [18]. In line with previous reports, scopolamine administration increased AChE activity while suppressing ChAT activity in the hippocampus, reflecting impaired acetylcholine availability [19, 20]. PPJM pretreatment dose-dependently normalized these alterations by reducing AChE activity and restoring ChAT activity, suggesting that modulation of the cholinergic system is a key mechanism underlying the cognitive benefits of PPJM. These biochemical changes are consistent with the observed behavioral improvements and support the role of PPJM in preserving cholinergic neurotransmission.

Beyond cholinergic regulation, synaptic plasticity and memory formation critically depend on neurotrophic signaling pathways, particularly the BDNF–TrkB–AKT–CREB axis [21, 22]. In the present study, scopolamine significantly suppressed hippocampal BDNF expression and TrkB phosphorylation, accompanied by reduced activation of downstream AKT and CREB signaling. PPJM pretreatment effectively counteracted these inhibitory effects, leading to a pronounced restoration of BDNF expression and TrkB activation, especially at the higher dose. The concomitant recovery of AKT and CREB phosphorylation suggests that PPJM promotes neuroplasticity-related transcriptional programs essential for learning and memory consolidation.

Tau hyperphosphorylation and aberrant activation of stress-responsive MAPK signaling pathways are closely associated with synaptic dysfunction and neurodegeneration [23, 24]. Consistent with this pathological profile, scopolamine administration markedly increased p-Tau and activated JNK and p38 MAPKs in the hippocampus. PPJM pretreatment significantly suppressed these molecular alterations, indicating that PPJM mitigates scopolamine-induced tau pathology and stress signaling. The inhibition of JNK and p38 activation may be particularly relevant, as these kinases are known to directly contribute to tau phosphorylation and neuronal dysfunction under oxidative and inflammatory conditions [25].

Neuroinflammation represents a critical pathological component of cognitive impairment, with NF-κB–mediated transcription serving as a central regulator of pro-inflammatory gene expression in the hippocampus [26]. Activation of NF-κB signaling promotes the transcription of multiple inflammatory mediators, including COX-2 and pro-inflammatory cytokines such as TNF-α and IL-6, which are known to impair synaptic function and neuronal survival [27]. In the present study, scopolamine administration markedly increased the protein expression of COX-2 and TNF-α and significantly enhanced NF-κB phosphorylation, indicating increased activation of neuroinflammatory signaling in hippocampal tissue. PPJM pretreatment significantly attenuated the scopolamine-induced upregulation of COX-2 and TNF-α, suggesting suppression of key inflammatory mediators mediators downstream of NF-κB activation. Given that COX-2 contributes to prostaglandin-mediated neuroinflammatory responses [28] and TNF-α is a potent cytokine implicated in synaptic dysfunction and memory impairment [29], the reduction of these factors by PPJM may be associated with the observed cognitive improvements. In contrast, IL-6 expression exhibited only a modest increase following scopolamine administration, and a statistically significant reduction was observed exclusively in the high-dose PPJM group. This differential response suggests that IL-6 regulation may require a higher threshold of anti-inflammatory intervention or may be governed by additional signaling pathways beyond NF-κB alone.

Collectively, these findings indicate that PPJM may exert anti-inflammatory effects in association with modulation of NF-κB signaling, associated with reduced expression of highly NF-κB–responsive targets such as COX-2 and TNF-α, while modulation of IL-6 appears to be more dose-sensitive. This selective cytokine regulation pattern highlights the complexity of neuroinflammatory signaling and suggests that PPJM may preferentially target dominant inflammatory drivers rather than uniformly suppressing all downstream cytokines. Histological analyses further supported the neuroprotective effects of PPJM. Scopolamine-induced neuronal disorganization and cellular shrinkage in the hippocampal CA1 region were markedly alleviated by PPJM pretreatment. In parallel, immunohistochemical staining demonstrated restoration of TrkB expression and suppression of p-Tau accumulation, suggesting preservation of neuronal integrity and modulation of key molecular targets implicated in cognitive function.

Previous studies have reported synergistic antioxidant and anti-inflammatory effects between propolis and *P. japonicus* in cellular models, suggesting potential complementary biological activities between the two components. In the present study, however, only the combined PPJM formulation was evaluated in the scopolamine-induced cognitive impairment model. Therefore, although the observed neuroprotective effects may be consistent with the previously reported complementary activities of these components, the current experimental design does not allow direct assessment of synergistic or additive interactions *in vivo*. Further studies including single-component treatment groups will be required to clarify the contribution of each component to the overall activity of PPJM.

It should be noted that the scopolamine model represents an acute pharmacological model of cholinergic dysfunction rather than a chronic neurodegenerative tauopathy. Although scopolamine administration has been reported to induce alterations in tau phosphorylation and neuroinflammatory signaling, these changes are generally considered to reflect transient stress-related signaling responses associated with cholinergic blockade. Therefore, the molecular alterations observed in the present study should be interpreted within the context of an acute memory impairment model rather than as direct evidence of disease-modifying effects in neurodegenerative disorders. Nevertheless, the ability of PPJM to modulate cholinergic activity, neurotrophic signaling, tau phosphorylation, and neuroinflammatory pathways suggests that it may influence key mechanisms involved in cognitive dysfunction.

## Conclusion

The present findings indicate that PPJM confers significant protection against scopolamine-induced memory impairment through a multifaceted mechanism involving restoration of cholinergic function, activation of BDNF–TrkB–AKT–CREB signaling, suppression of tau hyperphosphorylation and MAPK activation, and attenuation of neuroinflammatory responses. These results suggest that PPJM may modulate multiple molecular pathways associated with cognitive impairment. However, further studies using chronic neurodegenerative disease models will be necessary to clarify its long-term efficacy and therapeutic relevance.

## Supplemental Materials

Supplementary data for this paper are available on-line only at http://jmb.or.kr.
