# Supplementary material for: Protective Effects of a Propolis–Petasites japonicus Mixture on Scopolamine-Induced Memory Impairment in Mice
Source: J Microbiol Biotechnol. 2026 Apr 27;36:e2602019. doi: 10.4014/jmb.2602.02019 (PMC13146491; doi:10.4014/jmb.2602.02019)
Supplement: Supplementary file 1 [file jmb-36-e2602019-supple.pdf]

J. Microbiol. Biotechnol. 2026. 36: e2602019

https://doi.org/10.4014/jmb.2602.02019

Supplementary Table and Figures

Table S1. Quantification of marker compounds in PPJM determined by HPLC–DAD. Values represent the concentrations of pinocembrin, chrysin, and bakkenolide B in three independent production batches of PPJM. Data are expressed as mg/g of extract.

Batch	Pinocembrin (mg/g)	Chrysin (mg/g)	Bakkenolide B (mg/g)
Batch 1	0.0381	0.2496	1.0828
Batch 2	0.0361	0.2496	1.0708
Batch 3	0.0373	0.2499	1.0803
Mean	0.0372	0.2497	1.0781
SD	0.0010	0.0002	0.0063

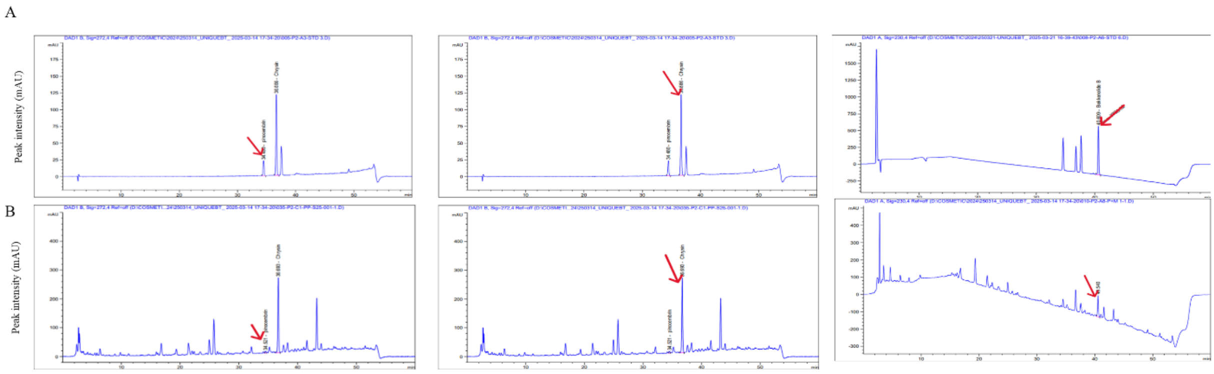

Fig. S1. HPLC chromatograms of PPJM and reference standards. (A) Chromatograms of the reference standards pinocembrin, chrysin, and bakkenolide B. **(B)** HPLC chromatogram of PPJM detected at 280 nm. The peaks corresponding to pinocembrin, chrysin, and bakkenolide B in PPJM were identified by comparison with the retention times of the reference standards using HPLC–DAD.

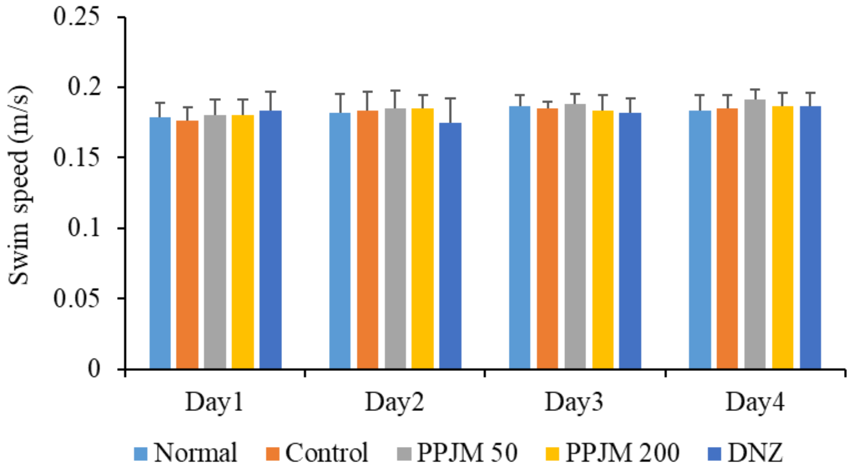

Fig. S2. Average swim speed during the acquisition phase of the Morris water maze test (Days 1–4). Swim speed was analyzed as an indicator of locomotor performance to evaluate potential non-cognitive factors influencing task performance. No significant differences in swim speed were observed among the experimental groups, suggesting that the differences in escape latency and path length were unlikely to be attributable to alterations in locomotor performance.

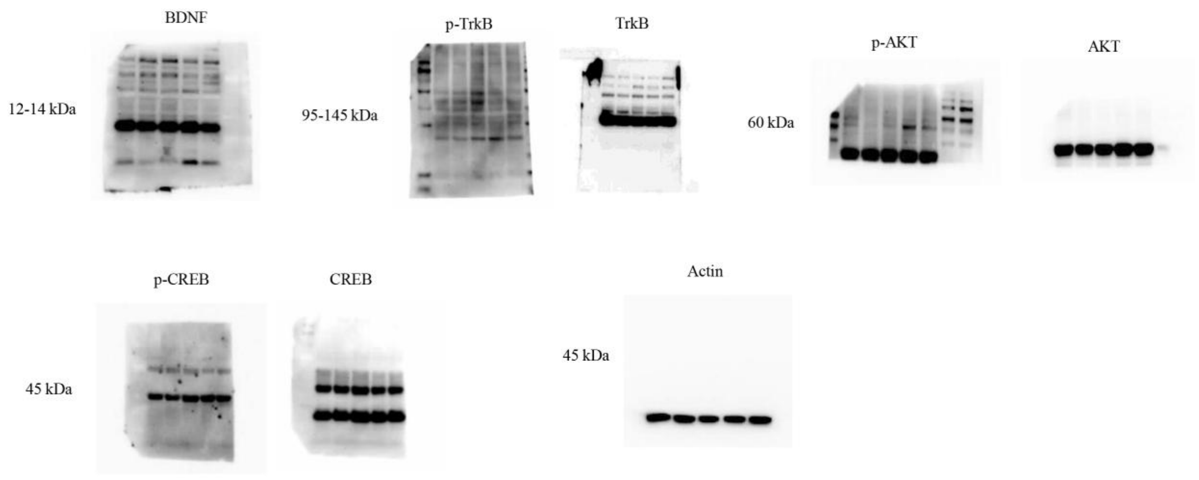

Fig. S3. Uncropped Western blot images used in Figure 3.

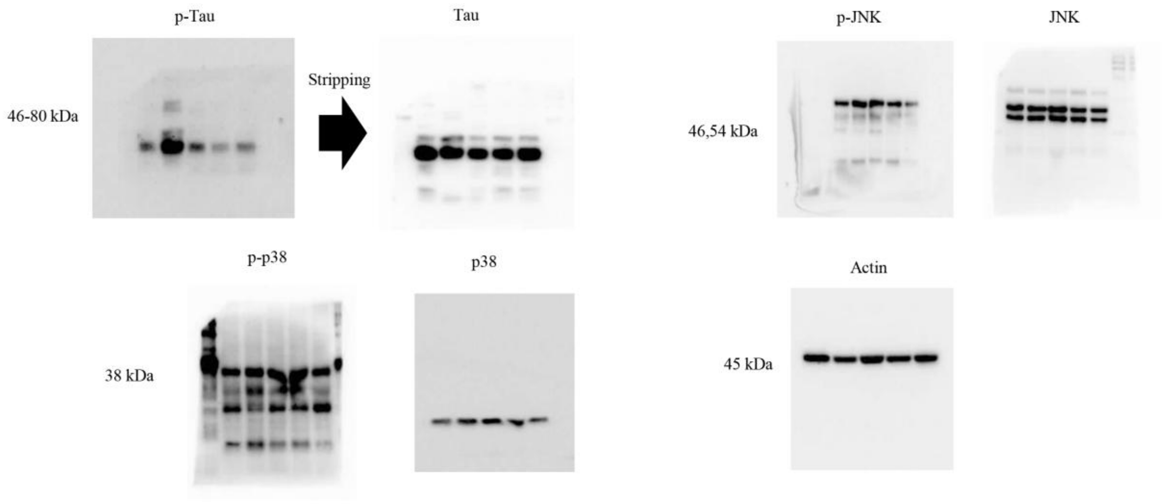

Fig. S4. Uncropped Western blot images used in Figure 4.

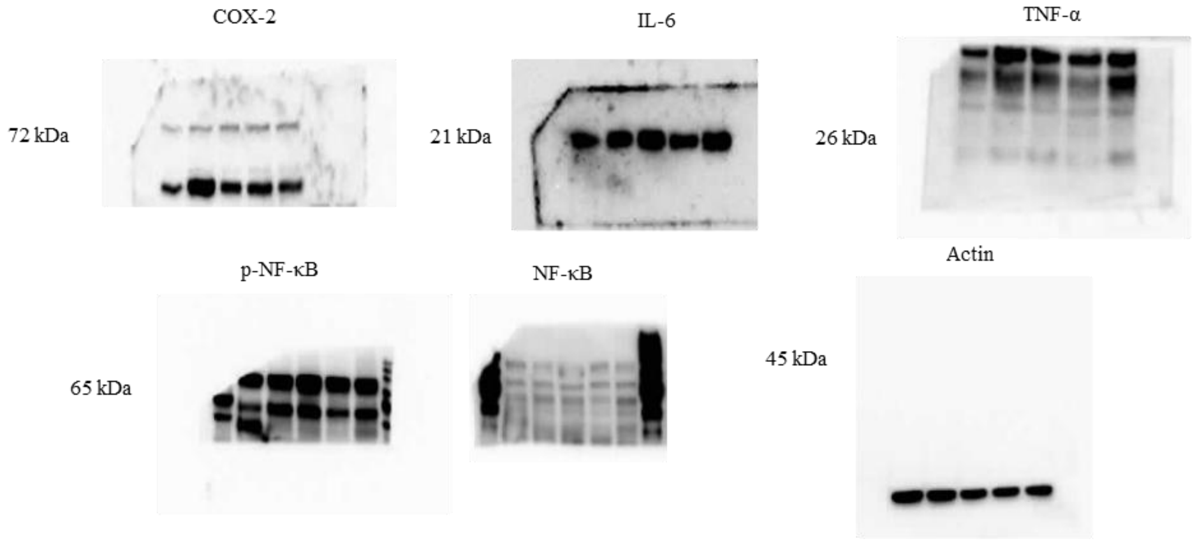

Fig. S5. Uncropped Western blot images used in Figure 5.
